# Comparative Studies on Electrodes for Rumen Bacteria Microbial Fuel Cells

**DOI:** 10.3390/s23084162

**Published:** 2023-04-21

**Authors:** Yusuke Yashiro, Michitaka Yamamoto, Yoshihiro Muneta, Hiroshi Sawada, Reina Nishiura, Shozo Arai, Seiichi Takamatsu, Toshihiro Itoh

**Affiliations:** 1 Department of Precision Engineering, Graduate School of Engineering, The University of Tokyo, 7-3-1 Hongo, Bunkyo-ku 113-8656, Tokyo, Japan; 2 National Institute of Animal Health, National Agriculture and Food Research Organization, 3-1-5 Kannondai, Tsukuba-shi 305-0856, Ibaraki, Japan

**Keywords:** cattle, rumen bacteria, microbial fuel cells, bamboo charcoal electrode, copper paper, power supply

## Abstract

Microbial fuel cells (MFCs) using rumen bacteria have been proposed as a power source for running devices inside cattle. In this study, we explored the key parameters of the conventional bamboo charcoal electrode in an attempt to improve the amount of electrical power generated by the microbial fuel cell. We evaluated the effects of the electrode’s surface area, thickness, and rumen content on power generation and determined that only the electrode’s surface area affects power generation levels. Furthermore, our observations and bacterial count on the electrode revealed that rumen bacteria concentrated on the surface of the bamboo charcoal electrode and did not penetrate the interior, explaining why only the electrode’s surface area affected power generation levels. A Copper (Cu) plate and Cu paper electrodes were also used to evaluate the effect of different electrodes on measuring the rumen bacteria MFC’s power potential, which had a temporarily higher maximum power point (MPP) compared to the bamboo charcoal electrode. However, the open circuit voltage and MPP decreased significantly over time due to the corrosion of the Cu electrodes. The MPP for the Cu plate electrode was 775 mW/m^2^ and the MPP for the Cu paper electrode was 1240 mW/m^2^, while the MPP for bamboo charcoal electrodes was only 18.7 mW/m^2^. In the future, rumen bacteria MFCs are expected to be used as the power supply of rumen sensors.

## 1. Introduction

Artificial intelligence, and subcategories such as machine learning and edge computing have developed rapidly in recent years, including their application in biosensors, wireless communication, and generators. [1,2,3]. In the field of livestock production, health monitoring of ruminants such as cattle and sheep using information technology has also been receiving attention [4,5,6,7,8,9]. Specifically, pH level monitoring of cattle’s rumen is critical because having significantly low pH levels in cattle’s rumen is strongly linked with rumen acidosis, a deadly condition that causes an overproduction of acid [10,11,12]. To monitor rumen pH levels, orally administered rumen sensors have been developed [13,14,15,16,17]; however, their short device lifespan of only a few months is insufficient for monitoring the entire livestock breeding period. Such a short device lifespan is mainly due to a power supply problem.

One approach to cultivate a longer lifetime power supply for rumen sensors is to generate power inside the rumen and drive the devices via the produced power. For example, Galvano batteries that use pH differences [18,19], energy harvesters that use vibration or thermal energy [20,21], and microbial fuel cells (MFCs) [22,23,24,25] have been proposed as methods for generating electrical power for the monitoring devices. Of these approaches, MFCs have attracted attention as a sustainable battery because they can theoretically be used semi-permanently as long as the conditions are right for the microorganisms and bacteria to persist [26,27].

In MFCs, the electron donor is oxidized under anoxic conditions by anaerobic bacteria through fermentation. The electron is then supplied to the anode via contact between the bacteria and the electrode or through nanowires or mediators [22,28]. The electron supplied to the anode electrode is transferred to the cathode electrode through a conductor, where it is reduced mainly by air or dissolved oxygen. In some cases, it can be reduced directly by bacteria. This electron reduction completes the electrical reaction, allowing MFCs to function as a power supply.

Recently, bacteria that can generate electrons have been discovered in the rumen of cattle [29], and rumen bacteria MFCs using bamboo charcoal electrodes have been suggested [30]. The bamboo charcoal electrodes used in previous studies have merits, such as being inexpensive, stable, and biocompatible because they are naturally occurring conductive materials. Furthermore, bamboo charcoal has a porous hexagonal structure and therefore may have a relatively large surface area. However, the power generated by rumen bacteria MFCs using bamboo electrodes was insufficient to drive the rumen sensors. Therefore, there is a need to improve power generation using bamboo charcoal electrodes or to explore other alternative electrode materials.

Generally, for many types of batteries, the surface areas and resistances of the electrodes influence power generation levels. Especially in MFCs, the number of bacteria adhering to the electrodes is important for power generation levels. Thus, the larger the electrode area and volume, the more power is expected to be generated. As for electrode resistance, the lower it is, the more power is expected to be generated because it is easier for electrodes to conduct electrons emitted from the bacteria. Based on the above estimates, this study evaluated the effects of the bamboo charcoal electrode structure, including surface area, thickness, and the gap between electrodes on power generation levels. Furthermore, other types of conductive electrodes, a Cu plate electrode and Cu paper, were investigated as electrodes for rumen bacteria MFCs. Cu paper is a conductive paper made of Cu fiber, thus providing it a large surface area and low electrical resistance, which is expected to result in high levels of power generation.

## 2. Materials and Methods

### 2.1. Effect of the Bamboo Charcoal Electrode Structure on Rumen Bacteria MFCs

**Figure 1:**
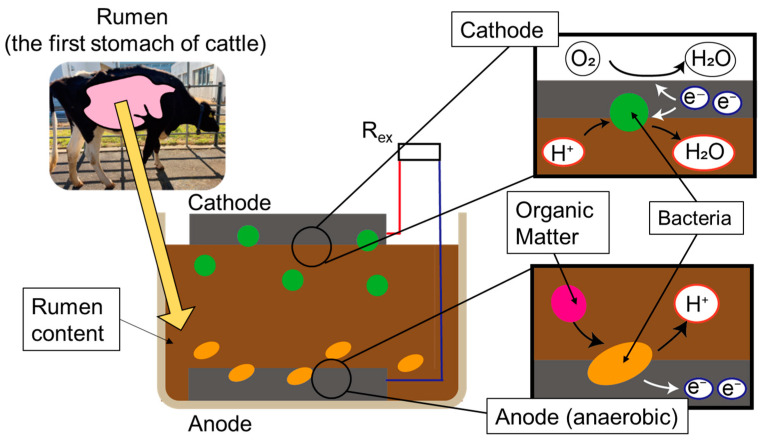
Image of the rumen bacteria MFC.

Rumen bacteria MFCs are fabricated by extracting rumen contents from the fistulated cow and layering them with electrodes in a container as shown in Figure 1. In the rumen of cattle, there are several types of bacteria, but after fabrication, anaerobic bacteria move to an anaerobic environment at the bottom of the case and supply electrons to the anode electrode. Electrons are transferred through a conductor, and bacteria reduce the electrons at the cathode side.

There are two parameters to consider in rumen bacteria MFCs using bamboo charcoal electrodes (Bamboo charcoal flat plate for commercial use, The Bamboo Charcoal Market, Fukuoka, Japan). The first parameter is the volume of rumen content. We hypothesized that the bacteria habitat conditions might change by adjusting the gap between the electrodes, which can lead to differences in power generation levels. The second parameter is the shape of the bamboo charcoal electrode, which is measured in two parts: the electrode’s surface area and the electrode’s thickness. Generally, increasing the surface area of MFCs increases the amount of electricity generated; thus, power generation levels will be improved by increasing the electrode’s surface area. Furthermore, if bacteria can penetrate into the interior of the bamboo charcoal electrode, it is expected that increasing the volume, in other words, increasing the electrode’s thickness, will also increase the amount of electricity generated. Therefore, this study evaluates the effect of the above three parameters on the rumen bacteria MFCs using a bamboo charcoal electrode.

Table 1 summarizes the experimental parameters using the bamboo charcoal electrode. To evaluate the impact of the bamboo charcoal electrode’s surface area on the MFCs power output (Condition 1), the rumen content’s thickness and the bamboo charcoal electrode’s thickness were set to 45 mm and 5 mm, respectively, while the electrode surface area was changed to 4900, 6700, 9400, and 13,900 mm^2^. To evaluate the impact of the bamboo charcoal electrode’s thickness on the MFCs power output (Condition 2), the rumen content’s thickness and the bamboo charcoal electrode’s surface area were set to 45 mm and 6700 mm^2^, respectively, while the thickness was changed to 3.5, 5, 7, and 9 mm. Finally, to evaluate the impact of the rumen contents thickness on the MFCs power output (Condition 3), the bamboo charcoal electrode’s thickness and surface area were set to 5 mm and 6700 mm^2^, respectively, while the rumen content’s thickness was changed to 10, 30, 45, and 60 mm. Notably, the bamboo charcoal used in this study was the general commercial-use bamboo charcoal, and the rumen contents were collected from fistulated cattle.

**Figure 2:**
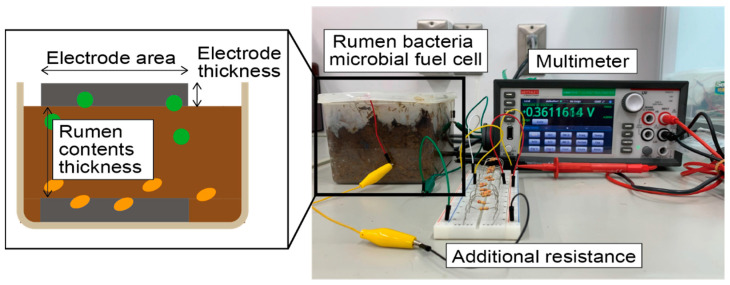
Image of the measurement setup. The relationship between each parameter is also shown.

To evaluate the generated power levels, a polarization curve was calculated by connecting the cells with an additional resistance varying between 50.9 Ω and 462,920 Ω and measuring the voltage using a digital multimeter (Keithley, DMM7510-900-02). Figure 2 depicts the measurement setup and the relationship between each parameter. The open circuit voltage (OCV) and maximum power point (MPP) were then evaluated from the calculated polarization curves. In rumen bacteria MFCs, the generated power may also depend on the number of bacteria and bacteria activities, which are difficult to control. Thus, evaluation was performed multiple times for each combination of conditions. Rumen contents collected simultaneously were used for the same timing measurement.

### 2.2. Observation and Bacterial Count on Bamboo Charcoal Electrodes

Since bacteria play an important role in rumen bacteria MFCs, it is important to clarify where bacteria are concentrated and the structures that tend to attract them. In this study, a scanning electron microscope (SEM, Hitachi S-4800, Hitachi, Ltd., Tokyo, Japan) was used to confirm the presence of bacteria on the bamboo charcoal electrode.

To evaluate the number of bacteria, concentrated on the electrode, the bamboo charcoal electrode was cut into three blocks two weeks after fabrication, and nine locations were assessed on each of the three surfaces, including the surface that had been in contact with the rumen contents prior to cutting. Each evaluation point was rubbed with a cotton swab, diluted with phosphate-buffered saline, and cultured onto a Petri dish with agar medium (Nissui Plate Sheep Blood Agar, NISSUI PHARMACEUTICAL CO., LTD., Tokyo, Japan) to increase the bacterial count. Because the growth of a single bacteria forms one colony, we can determine the number of bacteria by counting the number of colonies. However, it is important to note that this evaluation can only show the tendency of some bacteria, not the exact bacterial count.

### 2.3. Evaluation of Rumen Bacteria MFCs Using Cu Plate and Cu Paper Electrodes

**Figure 3:**
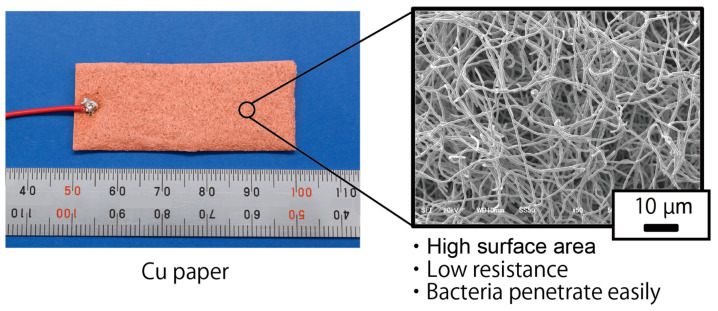
Image of the Cu paper.

In this study, to evaluate other material electrodes instead of a bamboo charcoal electrode for rumen bacteria MFCs, a Cu plate electrode (CU-113461, The Nilaco Corporation, Tokyo, Japan) and a Cu paper electrode fabricated by TOMOEGAWA CO., LTD. (Tokyo, Japan) was used instead of the bamboo charcoal electrode. The size of each electrode is 20 mm in width and 50 mm in length. The thickness of the Cu plate electrode is 0.5 mm, and the thickness of the Cu paper electrode is around 1.5 mm. Since the measured resistivity of Cu is approximately 1.5 × 10^−8^ Ω·m, which is low compared to that of the bamboo charcoal (around 286 Ω·m), the difference in electrode resistance can be evaluated by comparing the Cu plate electrode to the bamboo charcoal electrode. Furthermore, Cu paper is made of Cu fiber; which has a low electric resistance comparable to Cu plates and a perforated structure. Therefore, the effect of the perforated structure is expected to be determined by comparing the Cu plate and Cu paper electrodes. Figure 3 depicts the Cu paper electrode. Three cells were fabricated for each electrode evaluation. The evaluation method for each electrode was the same as the evaluation of the bamboo charcoal electrode.

## 3. Results

### 3.1. Effect of the Bamboo Charcoal Electrode Structure on Rumen Bacteria MFCs

**Figure 4:**
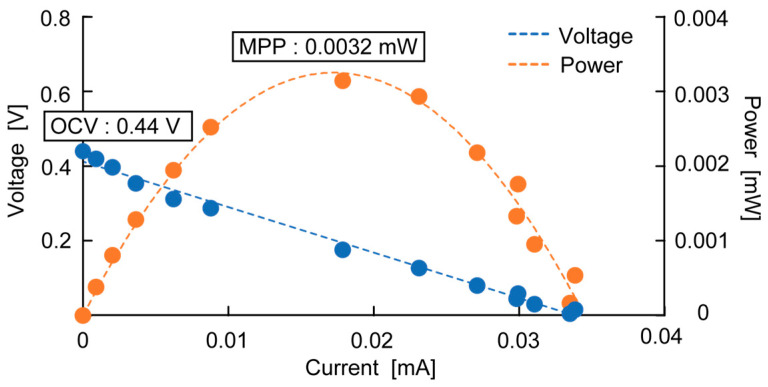
Typical measurement result of OCV and MPP. OCV is where the voltage is at its maximum, and MPP is where the power is at its maximum.

**Figure 5:**
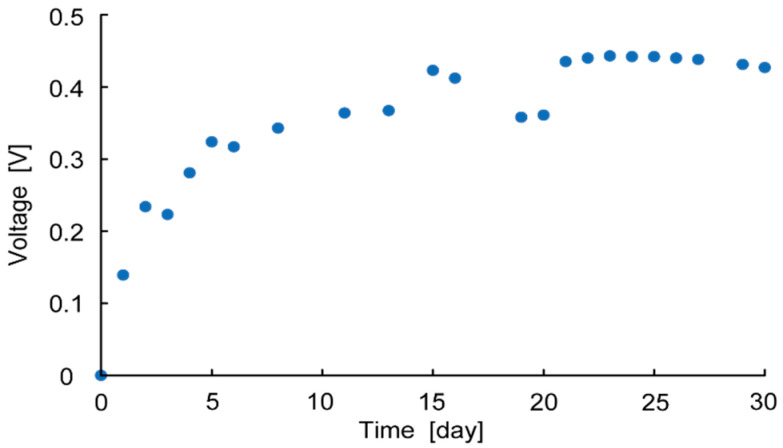
Measurement result of OCV over 30 days.

The typical measurement results of OCV and MPP are shown in Figure 4, with the OCV being recorded as the maximum voltage measured and the MPP being the maximum power measured from the electrode. Figure 4 shows the measurement results of a bamboo charcoal electrode with a thickness of 5 mm, an area of 6700 mm^2^, and a rumen content thickness of 45 mm. Figure 5 shows the measurement results of OCV over 30 days. This shows that the OCV increases over time and is saturated after two weeks, at which point the voltage plateaus. This is because it takes time for the bacteria to migrate to the anaerobic environment. Thus, OCV and MPP were measured two weeks after each cell fabrication to evaluate each parameter.

**Figure 6:**
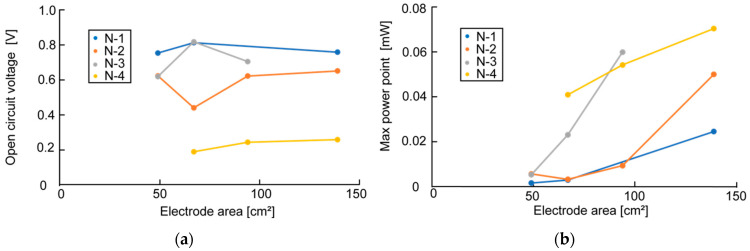
Measurement results of OCV and MPP in the experiment of changing the electrode area: (**a**) measurement result of OCV; (**b**) measurement result of MPP.

**Figure 7:**
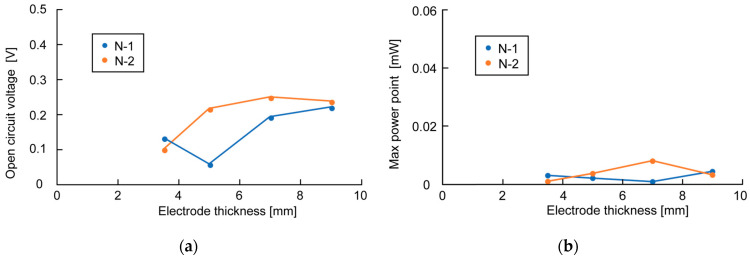
Measurement results of OCV and MPP in the experiment of changing electrode thickness: (**a**) measurement result of OCV; (**b**) measurement result of MPP.

**Figure 8:**
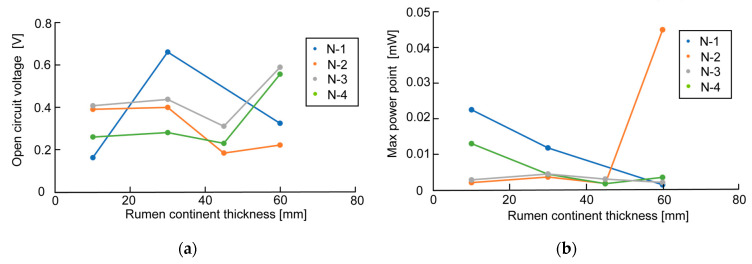
Measurement results of OCV and MPP in the experiment of changing rumen content thickness: (**a**) measurement result of OCV; (**b**) measurement result of MPP.

Figure 6, Figure 7 and Figure 8 show the measurement results of OCV and MPP when changing the bamboo charcoal electrode’s area, thickness, and rumen content thickness. Each label of the graphs indicates the timing of the measurement. As mentioned above, rumen contents collected simultaneously were used for the same timing measurement. Figure 6a shows that although the measured value varied with the timing of the measurement, the electrode area did not affect OCV. However, Figure 6b shows a clear trend between the electrode area and MPP, with MPP increasing as the electrode area increases. Figure 7 and Figure 8 show no influence of electrode thickness and the rumen content thickness on OCV and MPP, and thus no regularity in the change in values can be confirmed.

### 3.2. Observation and Counting Results of Bacteria on Bamboo Charcoal Electrodes

**Figure 9:**
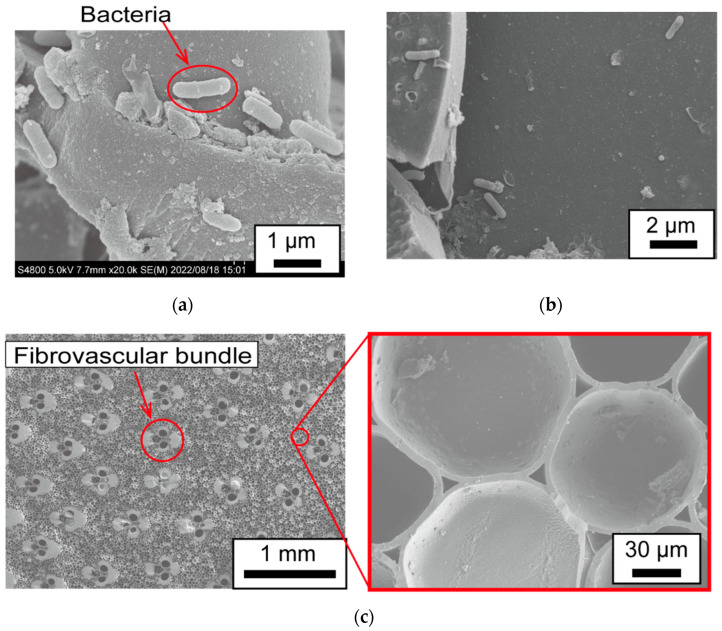
SEM observation results: (**a**) the bacteria on the surface of the bamboo charcoal electrode; (**b**) the interior of the bamboo electrode where there are few bacteria; and (**c**) the honeycomb structure inside the bamboo charcoal.

Figure 9 depicts the SEM observation results. Figure 9a is the SEM image of the surface of the bamboo charcoal electrode, and it indicates that bacteria of about 1.5 μm in diameter were present on the surface of the bamboo charcoal electrode. These bacteria seem to be locally clumped rather than sparsely distributed, indicating that biofilms are formed. Figure 9b shows the SEM observation results of the interior of the bamboo charcoal electrode. Figure 9b shows fewer bacteria in the interior than on the surface of the bamboo charcoal electrode. This is because, as shown in Figure 9c, the honeycomb structure inside the bamboo charcoal had no holes penetrating it longitudinally except for the vascular bundles, making it difficult for bacteria to move inside the bamboo charcoal electrode.

**Figure 10:**
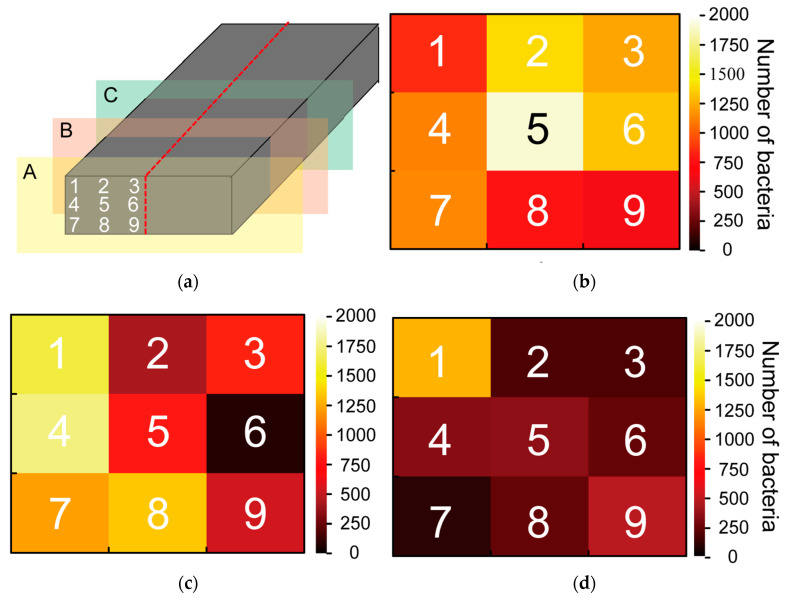
Evaluation results of the number of bacteria on the bamboo charcoal electrode: (**a**) the observation point; (**b**) the surface of the bamboo charcoal electrode (cross-section A); (**c**) 2nd cross-section of the bamboo charcoal electrode (cross-section B); and (**d**) interior of the bamboo charcoal electrode (cross-section C).

Figure 10 depicts the evaluation results of the bacterial count on the bamboo charcoal electrode. Figure 10a shows the observation points. A large number of bacteria are present on the surface (Figure 10b), and the number of bacteria decreases as one moves toward the interior of the bamboo charcoal (Figure 10c,d). Moreover, inside the bamboo charcoal, the number of bacteria is higher where the bamboo charcoal is in contact with its surroundings. (Figure 10c,d). These results suggest that there are more bacteria on the surface of the bamboo charcoal electrode, and bacteria do not enter the interior.

However, it should be noted that although we observed and evaluated the number of bacteria in this study, we cannot conclude whether the observed and counted bacteria generate electricity because the bacteria responsible for generating electricity remains unelucidated.

### 3.3. Experiment Result of Rumen Bacteria MFCs Using Cu Plate and Cu Paper Electrodes

**Figure 11:**
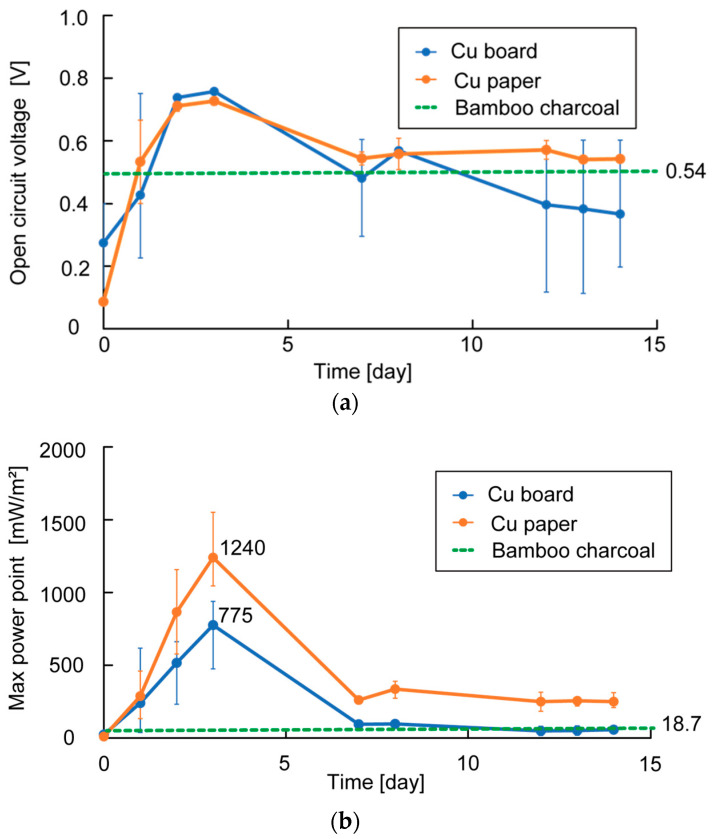
Measurement results of OCV and MPP of each electrode: (**a**) measurement result of OCV; (**b**) measurement result of MPP.

Figure 11 depicts the OCV and MPP measurement results for each Cu plate electrode and Cu paper electrode. Each value represents the average of the three cells. The result of the highest OCV and MPP in the case of using bamboo charcoal electrodes are also shown in Figure 11. The figure also shows that the OCV of using Cu plate electrodes and Cu paper electrodes were equal, and their highest OCV was higher than that of using bamboo charcoal electrodes. The measured highest MPP of using the Cu plate electrode was 775 mW/m^2^ and the measured highest MPP of using the Cu paper electrode was 1240 mW/m^2^, while that of using the bamboo electrode was only 18.7 mW/m^2^. This indicates that using electrodes with low resistance can improve the OCV and MPP.

Furthermore, comparing the highest MPP of the Cu plate and Cu paper electrodes, the perforated structure-like Cu paper electrodes appear to be more effective in improving power generation. However, the OCV and MPP decreased significantly over time when using Cu plate electrodes and Cu paper electrodes. This may be due to the corrosion of Cu over time, as both electrodes largely darkened and oxidized on the surface.

## 4. Discussion

In this study, we investigated the effects of the area and thickness of the bamboo charcoal electrode and the thickness of the rumen contents of rumen bacteria MFCs. The effects of the electrode resistance were also evaluated by changing the electrode type. The result of the relationship between these parameters and the OCV or MPP is summarized in Table 2. Table 2 also illustrates that only the electrode resistance was related to OCV, while the electrode area and resistance were related to MPP. The higher electrode area resulting in higher power generation levels is thought to be due to the increased number of bacteria attached to the electrodes, which in turn increases the amount of current.

Next, we discussed the effects of the area and thickness of the bamboo charcoal electrode. During these experiments, changing the electrode thickness of the bamboo charcoal electrode significantly changed the volume compared to the surface area. Thus, power generation is expected to be improved if the bacteria can penetrate the interior of the bamboo charcoal electrode. However, the experimental results show that changing the electrode thickness has no effect on power generation levels. Furthermore, observation and the bacterial count on the bamboo charcoal electrode revealed that bacteria only live on the surface of the bamboo charcoal electrode rather than the interior. Therefore, increasing the thickness of the bamboo charcoal electrode does not increase the number of settled bacteria. These results indicate that an electrode with a large surface area and structure, which bacteria can easily penetrate, is essential in improving power generation. Additionally, bamboo charcoal electrodes require alterations to their surface area to facilitate bacterial penetration, making them suitable for rumen bacteria MFCs.

During the experiment using Cu paper electrodes, the MPP improved significantly and exceeded 1000 mW/m^2^. This is because the copper paper has low resistance and a large surface area and structure, which bacteria can easily penetrate. To drive the rumen sensor, greater than 1 mW is required. Considering the size of the rumen sensor, the required power output by rumen bacteria MFCs is over 600 mW/m^2^. This indicates that the obtained value (over 1000 mW/m^2^) in the case of the Cu paper electrodes can drive the rumen sensor in the future, although there are problems, such as corrosion.

Furthermore, by developing an electrode with lower resistance and a larger surface area, it is expected that a larger MPP can be generated in the future. Note that in the experiment using Cu paper electrodes, the electrodes corroded after a certain period, resulting in decreased power generation. Thus, a method to prevent electrode corrosion should be developed in the future.

It should also be mentioned that research has been conducted where MFCs generate over 2800 mW/m^2^ using other types of bacteria and electrodes [31,32]. Although direct discussion of the present results is difficult due to the use of different types of bacteria, these previous studies show that further improvement in power generation levels is possible by further analysis of rumen bacteria.

## 5. Conclusions

We explored the key parameters for improving the amount of electrical power generation levels of rumen bacteria MFCs using bamboo charcoal electrodes and found that a larger electrode surface area is important for improving power generation levels. Furthermore, observations and bacterial counts on the bamboo charcoal electrode revealed that the electrode’s surface area has a relationship with power generation because rumen bacteria concentrate on the surface of the bamboo charcoal electrode due to being unable to penetrate into the interior.

Furthermore, by evaluating the power generation levels of the Cu plate electrode and Cu paper electrode instead of the bamboo charcoal electrode, the OCV and MPP were improved. MPP in the case of using Cu plate electrodes was 775 mW/m^2,^ and MPP in the case of using the Cu paper electrode temporarily exceeded 1000 mW/m^2^, while that of the bamboo electrode was 18.7 mW/m^2^. The MPP using the Cu paper electrode improved compared to that of the Cu plate electrode because the Cu paper electrode has a larger surface area and structure, which bacteria can easily penetrate. The obtained MPP of over 1000 mW/m^2^ can drive the rumen sensor, and rumen bacteria MFCs are expected to be used as the power supply of rumen sensors in the future. Since the electrodes corroded after a certain period and resulted in decreased power generation, a method to prevent electrode corrosion is required to extend the lifespan of power generators and realize their practical uses as a power source.

## Figures and Tables

**Table 1:** Experiment parameters in the case of changing the electrode’s surface area (Condition 1), electrode thickness (Condition 2) of bamboo charcoal electrodes, or rumen content’s thickness (Condition 3).

	Electrode Surface Area (mm^2^)	Electrode Thickness (mm)	Rumen Contents Thickness (mm)
Condition 1	4900, 6700, 9400, 13,900	5	45
Condition 2	6700	3.5, 5, 7, 9	45
Condition 3	6700	5	10, 30, 45, 60

**Table 2:** Summary of the relationship between experimented parameters and the OCV and MPP. (◯ means there is a correlation and × means there is no correlation).

	Electrode Area	Electrode Thickness	Rumen Contents Thickness	Electrode Resistance
OCV	×	×	×	◯
MPP	◯	×	×	◯

## Data Availability

Not applicable.
